# On the Quantum Confinement Effects in Ultrathin PdO Films by Experiment and Theory

**DOI:** 10.3390/ma15238700

**Published:** 2022-12-06

**Authors:** Alexandros Barnasas, Christos S. Garoufalis, Dimitrios I. Anyfantis, Panagiotis Poulopoulos, Sotirios Baskoutas

**Affiliations:** 1 Materials Science Department, University of Patras, 26504 Patras, Greece; 2 Institut für Physikalische Chemie, Universität Hamburg, Grindelallee 117, 20146 Hamburg, Germany

**Keywords:** thin films, PdO, optical properties, quantum confinement, potential morphing method

## Abstract

Radio frequency magnetron sputtering conducted in a high vacuum with a base pressure of $1\times 10^{-6}$ mbar was used to deposit ultrathin palladium films on Corning glass. The thickness of these films ranged from 0.4 to 13 nanometers. PdO films were produced after being post-annealed in a furnace at temperatures of 530 degrees Celsius in the presence of air. The results of an atomic force microscopy study showed that the material possessed a high crystalline quality with a low roughness. When looking at Tauc plots to determine the position of the direct optical band gap, the thicker films show a value that is relatively close to 2.2 eV. When the film thickness was reduced all the way down to 0.7 nm, a significant “blue shift” of more than 0.5 eV was observed. In order to provide a more in-depth understanding of the experiment, theoretical calculations based on the Hartree–Fock approximation as applied to an electron-hole system were performed in the framework of the effective mass approximation. The findings are regarded as empirical proof of the existence of quantum confinement effects.

## 1. Introduction

The crystal structure of palladium oxide is tetragonal; its space group is $D_{4h}^{9}$, and its a and c lattice constants are 0.30434 and 0.53363 nanometers, respectively [1]. In keeping with the characteristics of a d8 metal ion, the palladium atoms have a square planar shape. To a close approximation, oxygen atoms assume tetrahedral positions. PdO nanoparticles and thin films have recently attracted a lot of interest due to the possibility that they can be used in a wide variety of innovative applications.

These primarily include gas sensing [2,3,4,5,6,7,8,9] applications, as well as photoelectronic [10], photocatalytic [11], and plasmonic [12] applications. PdO is an important catalytic material in its own right. It is a p-type semiconductor with a direct band gap (due to a deviation from complete stoichiometry). According to various reports, the band gap value ranges between 0 and 2.67 eV [13]. It is worth noting that many theoretical studies report a zero-band gap, implying metallic behaviour [1,13]. Experimenters such as Rey et al., on the other hand, recorded an intense optical absorption spectrum with an onset after 1.7 eV. They reported a direct optical band gap of 2.13 eV measured by optical transitions and a value of 2.67 eV measured by photoconductivity measurements [14]. This difference was attributed to the fact that the first optical absorption peak at about 2.5 eV could be caused by excitonic transitions. At about 3.3 eV [14], a second broader peak appeared in the optical absorption spectrum. Nilsson et al. published their own relevant data, which agree with those in Refs. [14,15].

In this work, as part of our investigation into the effects of quantum confinement, we have been recording the optical absorption spectra of PdO. The annealing of palladium films at 530 degrees Celsius resulted in the production of ultrathin PdO films. By employing Tauc plots, we were able to determine the PdO absorption edge, (i.e., the direct optical band gap). As the film thickness decreases, we probe a significant increase in the position of the optical band gap (blue shift). This dependence of the optical band gap value on the thickness of the considered film is highly suggestive of a manifestation of quantum confinement effects.

The experimental results are supported by theoretical calculations based on a two-particle (electron and hole) Hartree Fock (HF) approximation suitably applied within the context of effective mass approximation (EMA). This approximation was chosen not only because the size of the systems under study is simply too large for more robust and advanced approximation (e.g., DFT), but also because a comprehensive microscopic treatment has difficulty capturing the quantum confinement effect (owing to passivation issues). These difficulties can be overcome by resorting to the reliable and well-tested combination of HF and EMA [16,17], which has a notable track record of efficient qualitative and quantitative treatments of such problems [18]. The numerical solution of the pertinent equations is obtained using the potential morphing method (PMM). Our theoretical conclusions for the dependence of PdO’s direct optical band gap on film thickness compare well with the experimental measurements. This agreement confirms that the observed changes may be safely attributed to quantum confinement effects.

## 2. Materials and Methods

### 2.1. Experimental Details

Ultrathin PdO films with thicknesses between 0.7–22 nm were produced after 60 min annealing of metallic Pd films at 530 °C in air. According to Ref. [4], annealing of Pd films 5–40 nm thick in such temperatures resulted in the formation of homogeneous PdO films. The ultrathin PdO films have a uniform light orange color, see also [14]. The Pd films were grown on Corning glass by radio frequency (r.f.) magnetron sputtering. A two-inch broad cylindrical target of Pd with a purity of 99.99 at. %, was mounted on the Torus 2 HV circular sputtering source of Kurt J. Lesker Company. The films were deposited at room temperature. The base pressure of the vacuum chamber was $1\times 10^{-6}$ mbar. The Ar partial pressure during deposition was about $1\times 10^{-2}$ mbar. The morphology of a selected sample was recorded by atomic force microscopy (AFM). The AFM used was a multimode microscope with a Nanoscope IIIa controller and a 120 μm × 120 μm magnet-free scanner (model AS-130VMF) developed by Bruker (Santa Barbara, CA, USA). The microscope was operated in the non-contact (tapping) mode [19].

In order to determine the film thickness, we scratched the surface of a film with a sharp tool. Then, we mounted the film on the AFM and measured the profile of this scratch, see, e.g., [20,21]. We measured the depth of this scratch at several points and took the average value. This was the film thickness determined with an accuracy of ±5%. A quartz balance system (Inficon XTM/2) was calibrated with the help of the previous measurement. After this, one can measure the films thickness with an accuracy of ±0.1 nm.

Finally, the ultraviolet UV-Vis measurements were carried out at room temperature in transmission geometry using a Shimadzu Uv-Vis Spectrophotometer, Model: UV 1800 (Shimadzu, Kyoto, Japan) at wavelengths ranging from 200 to 1100 nm.

### 2.2. Theory

The thin films are modeled by a two particle system consisting of one electron and one hole. The corresponding Hamiltonian is

(1) $$\hat{H}=-\frac{\hbar^{2}}{2m_{h}^{*}}\nabla_{h}^{2}-\frac{\hbar^{2}}{2m_{e}^{*}}\nabla_{e}^{2}+V_{0}^{h}\left(r_{h}\right)+V_{0}^{e}\left(r_{e}\right)-\frac{e^{2}}{\epsilon }\frac{1}{r_{he}}$$

where $m_{h}^{*}$ and $m_{e}^{*}$ are the effective masses of the hole and the electron, respectively. For the model to produce meaningful results, it is necessary to take into consideration the dependence of the dielectric constant ϵ (present in the last term of the Hamiltonian) on the thickness of the film. This is achieved by adopting the approximations described in reference [22]. The term $V_{0}^{\left(h\right)}\left(r_{\left(h\right)}\right)$ corresponds to the confining potential of the hole, while the term $V_{0}^{\left(e\right)}\left(r_{\left(e\right)}\right)$ corresponds to the confining potential of the electron.

$$V_{0}^{e(h)}=\left\{\begin{array}{cc} 0 & |z|<L/2\\ V_{0} & |z|\geq L/2 \end{array}\right.$$

with $V_{0}$ the height of the well which is empirically determined [23]. The derived HF equations take the form

(2) $$\left[\frac{p_{\left(h,e\right)}^{2}}{2m_{\left(h,e\right)}^{*}}+U_{\left(h,e\right)}\left(r_{\left(h,e\right)}\right)\right]\Phi_{\left(h,e\right)}\left(r_{\left(h,e\right)}\right)=E_{\left(h,e\right)}\Phi_{\left(h,e\right)}\left(r_{\left(h,e\right)}\right)$$

where the potential term *U* besides the confining terms referenced above. It also contains the exchange and the Coulomb interactions between the two considered particles (i.e., hole and electron). In particular, the action of *U* on the electron or hole wavefunctions is given by the equations

$$U_{e}\left(r_{e}\right)\Phi_{e}\left(r_{e}\right)=\left[V_{0}^{e}\left(r_{e}\right)-\frac{1}{2}\frac{e^{2}}{\varepsilon }\int dr_{h}\frac{|\Phi_{h}\left(\left.r_{h}\right)\right.{|}^{2}}{\left|r_{e}-r_{h}\right|}\right]\Phi_{e}\left(r_{e}\right)+\frac{1}{2}\frac{e^{2}}{\varepsilon }\int dr_{h}\frac{\Phi_{h}^{*}\left(r_{h}\right)\Phi_{e}\left(r_{h}\right)}{\left|r_{e}-r_{h}\right|}\Phi_{h}\left(r_{e}\right)$$

and

$$U_{h}\left(r_{h}\right)\Phi_{h}\left(r_{h}\right)=\left[V_{0}^{h}\left(r_{h}\right)-\frac{1}{2}\frac{e^{2}}{\varepsilon }\int dr_{e}\frac{|\Phi_{e}\left(\left.r_{e}\right)\right.{|}^{2}}{\left|r_{h}-r_{e}\right|}\right]\Phi_{h}\left(r_{h}\right)+\frac{1}{2}\frac{e^{2}}{\varepsilon }\int dr_{e}\frac{\Phi_{e}^{*}\left(r_{e}\right)\Phi_{h}\left(r_{e}\right)}{\left|r_{h}-r_{e}\right|}\Phi_{e}\left(r_{h}\right)$$

Although the solution of Equation (2) is achieved iteratively (as expected from basic HF theory) in the current implementation, the calculations of energy and wavefunctions at each iteration is accomplished by the PMM method. A detailed presentation of the approximations and the algorithms used throughout the implementation of the method can be found in Refs. [24,25].

After the numerical solution has been obtained, the exciton total energy is calculated by the expression $E\left(X\right)=E_{h}+E_{e}$, and the material’s effective gap is determined by the relation $E_{g}^{eff}=E_{g}^{bulk}+E\left(X\right)$.

## 3. Results and Discussion

**Figure 1:**
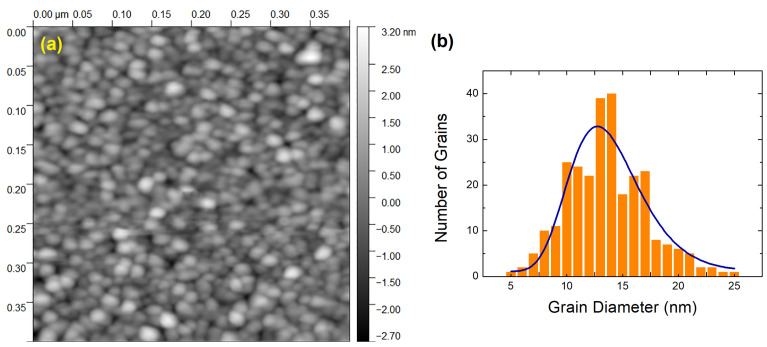
(**a**) AFM image recorded on the surface of an 8.9 nm thick PdO film. The roughness scale is introduced on the right-hand side of the image. In (**b**), the corresponding statistics of the grain size together with log-normal fitting is provided.

One can examine the surface topography of a PdO layer that is 8.9 nm thick by looking at Figure 1a. AFM was utilized in order to record the image. The film’s surface was analyzed for the presence of nanocrystallites. On the right-hand side of the image, the roughness scale is presented to the reader. The roughness of the surface is not particularly severe. Its root mean square value (RRMS) is only 1.4 nm. This figure suggests that the film has a generally flat surface. Palladium is known to operate as a surfactant and to assist in the creation of flat layers (for more information, consult references such as [26]). The grains are quite homogeneous in terms of size. The grain diameter (D) size distribution of the film of Figure 1a is depicted in Figure 1b. A log-norm function provides a satisfactory fit to the distribution [27]. The peak on the log-normal curve occurs at D = 12.7 nm. A relatively restricted size distribution can be deduced from the fact that the full width at half-maximum, or FWHM, is only 7.5 nm. Our AFM data characterize the PdO surface in the nanometer range. For an atomic level understanding of the surfaces of Pd and other transition metal oxides, see [28] and references therein.

**Figure 2:**
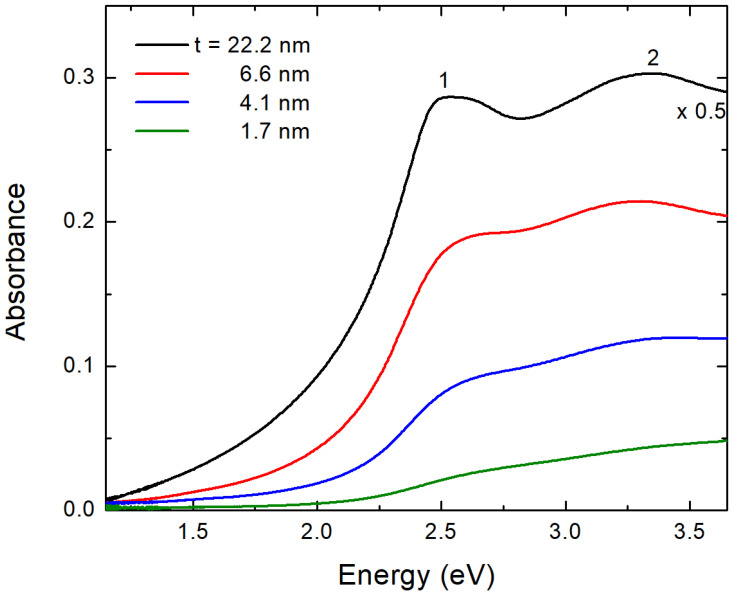
Absorbance spectra for four PdO films; their thickness t is indicated. The absorbance of the thickest film is multiplied by 0.5 for better presentation of the Figure.

In Figure 2, we plot the optical density (or absorbance A=−logT, where *T* is the transmittance) spectra [29] for four PdO films with the thickness t as indicated. The spectra are only shown for energies up to 3.7 eV. Beyond this threshold, Corning glass begins to absorb intensively and dominates the spectrum. However, because the optical band gap in PdO is only a little bit higher than 2 eV, this limitation does not affect our analysis. The spectrum of the 22.2 nm film has two distinct features that can be identified at 2.5 eV (designated as “1”) and 3.3 eV (designated as “2”) [14]. These characteristics can be thought of as fingerprints of properly grown polycrystalline PdO films. Both of these features, particularly the first one, become less noticeable as the thickness of the film is reduced. This could be an indication that the films have partially undergone amorphization [29,30], which occurs when the thickness of the film decreases significantly.

**Figure 3:**
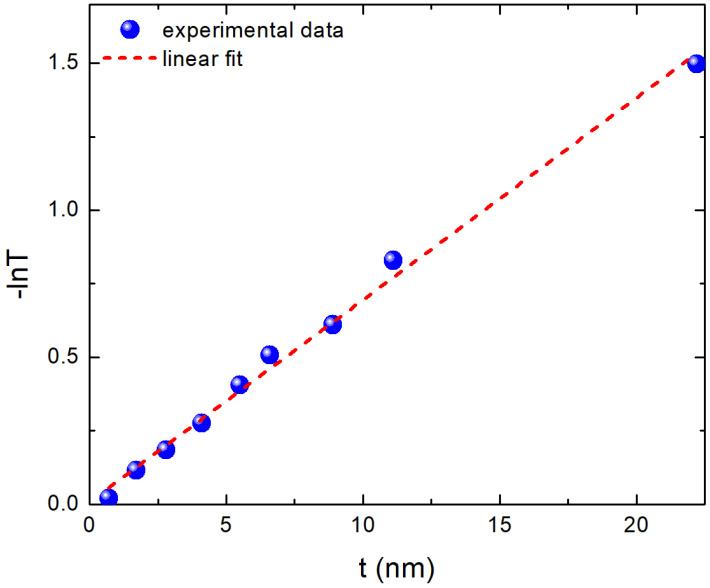
(−lnT) as a function of film thickness t. The slope of the linear fit is equal to the absorption coefficient α at the second feature of the absorbance spectra.

Before moving on to the Tauc plots for the evaluation of the optical band gap, we present in Figure 3 a plot of −lnT versus *t* in order to determine the absorption coefficient α of the samples. The slope of the plot in Figure 3 is used to derive the value of the absorption coefficient. We use the second feature of the absorbance spectra for this process because it is simpler to locate than the first feature; in fact, the first feature smoothens out more intensively, and it can be difficult to be clearly identified for the thinnest samples. The value of α is found to be α∼$7\times 10^{5}$ cm${}^{-1}$ when analyzed using the plot of Figure 3. This value is in good agreement (within about 10%) with the findings of the works cited in References [31,32].

**Figure 4:**
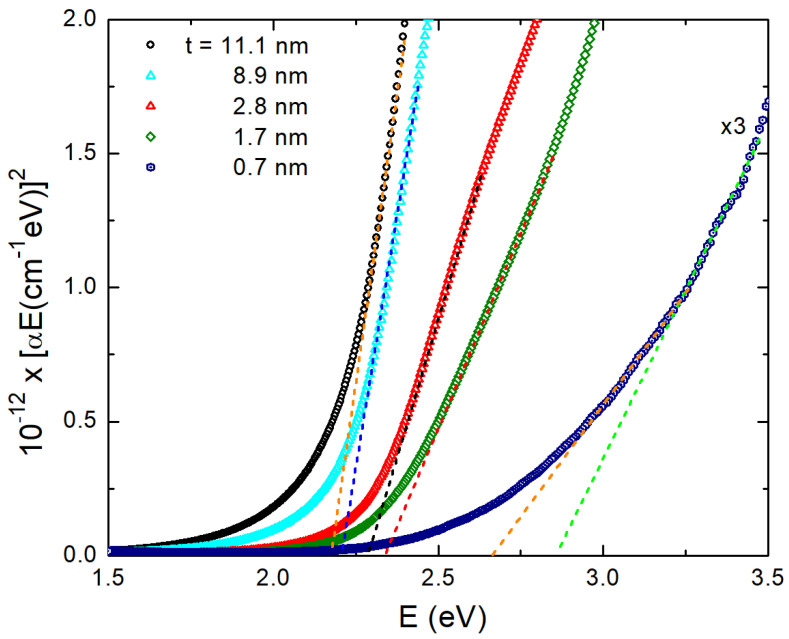
Tauc plots ((${\alpha E)}^{2}$ as a function of E) for five PdO films; their thickness t is indicated in the legend. One can clearly observe a “blue” shift of the energy gap as t decreases.

In Figure 4, we provide Tauc plots for the determination of the position of the direct optical band gap of our ultrathin PdO films. These are plots of the ${\left(\alpha E\right)}^{2}$ as a function of energy E, appropriate for a direct band gap semiconductor. The intercept of the linear part of the plot with the energy axis determines the position of the energy gap. One may see that there is a good linear part in the Tauc plots, and extrapolating to cross the X-axis provides the energy gap with an accuracy of about ±2 meV. Only for the case of the thinnest film is there some larger error bar. For the specific sample, two extreme lines are provided. The first of them gives an energy gap of about 2.66 eV, while the other one shows a gap of 2.85 eV. Therefore, the error bar is of about ±100 meV for the thinnest sample. We clearly observe a “blue shift” in the position of the optical band gap as the film thickness t decreases. This is better viewed in Figure 4: we show the results of the Tauc plots for the energy gap of PdO films. One may clearly see an energy increase as the film thickness decreases. Indeed, this may be observed if one goes to the thinner films where quantum confinement effects may be of importance as the film thickness becomes comparable to the Bohr exciton radius.

**Figure 5:**
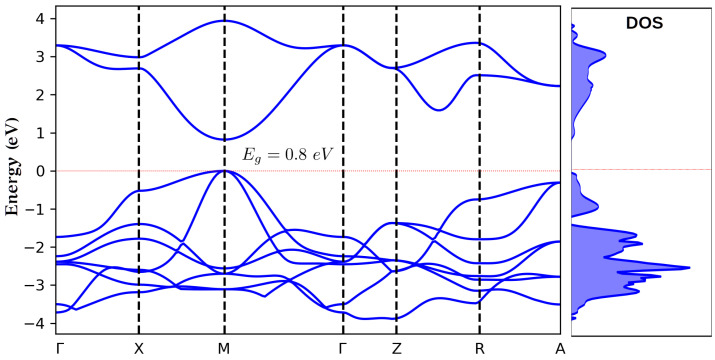
Band structure and density of states diagrams of PdO, as obtained by DFT/B3LYP/BOP-TZVP calculations.

In an effort to further investigate the observed behavior, we have additionally performed theoretical calculations (with the method described above) on samples of similar thicknesses as the ones reported by the experiments and compared their predictions with the presented experimental findings. The first necessary step before any potential morphing (PMM) calculation is to provide the electron and hole effective masses ($m_{e}^{*},\;m_{h}^{*}$), the dielectric constants ($\varepsilon_{0},\;\varepsilon_{\infty }$), and $\omega_{LO}$. However, in the present case, despite the extended literature search, we did not manage to find any values for the required masses of PdO. For this reason, we performed our own band-structure calculations based on density functional theory as a means to derive the values of $m_{e}^{*}$ and $\;m_{h}^{*}$. In particular, we employed the CRYSTAL06 [33] program, which is a code capable of performing calculations using periodic boundary conditions (PBC) with Gaussian basis sets. The calculations were performed with the hybrid exchange correlation functional B3LYP [34] with the BOP-TZVP basis sets [35,36]. The choice of a hybrid functional was dictated by the fact that all the existing GGA/DFT calculations on PdO predict a metallic behavior. This can be readily attributed to the intrinsic tendency of the GGA functionals to significantly underestimate the band gap. This tendency is usually partially remedied by including in the functional some percentage of exact exchange, as in the so-called hybrid functionals. In this context, B3LYP has proved quite successful in predicting band gaps [37,38,39,40,41]. The resulting band structure and the corresponding density of states (DOS) diagrams are presented in Figure 5. As is clearly shown, the use of a hybrid functional leads to a direct band gap located at the *M* point. This is found to be in complete agreement with early calculations [42] which also reached the same conclusion with regard to the position of the direct gap. However, the estimated value was found to be only ∼0.1 eV [42]. In our case, the hybrid B3LYP/BOP-TZVP calculation produced a significantly larger gap of 0.8 eV. This value, regardless of its accuracy, verifies the semiconducting nature of the material, the direct nature of the gap, and its position in *M* point. Moreover, the shape of the bands, which is critical for the determination of the hole and electron masses, usually does not depend strongly on the adopted functional and appears to be quite reliable even for calculations where the gap value is not accurately reproduced.

**Figure 6:**
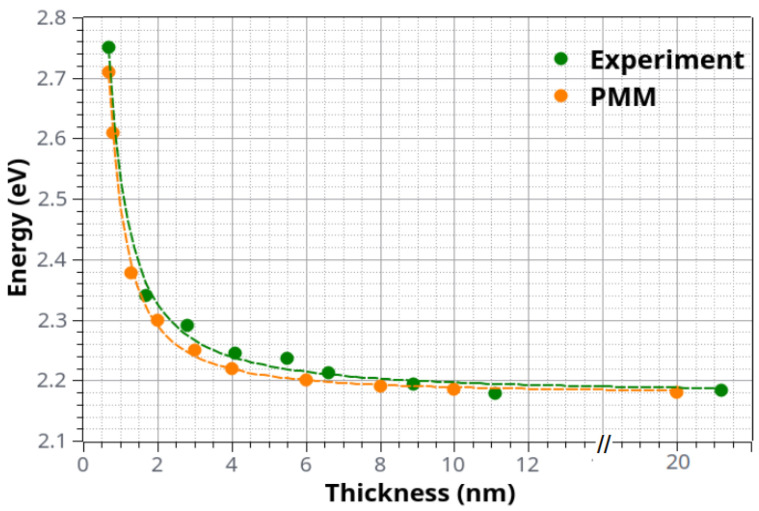
Direct optical band gap energy of ultrathin PdO films as a function of film thickness *t* by experiment and theory. The typical error bar is ±2 meV except in the case of the thinnest sample where it is about 50 times larger (see the two straight lines for this sample in the Tauc plots of Figure 4).

Following this argument, we used the CBM and VBM curves of the band structure diagram (Figure 5) in order to obtain the required effective masses for the hole and the electron ($m_{h}^{*}=2.27,\;m_{e}^{*}=1.87$), while the corresponding values of $\varepsilon_{0},\;\varepsilon_{\infty }$, and $\omega_{LO}$ were taken from the literature [15,43]. With these parameters fixed, a series of calculations were performed (using the two-particle Hamiltonian described in Section 2.2) for sample thicknesses relevant to the ones considered in the experiments. The results are presented in Figure 6 alongside the experimental ones. It is evident that the agreement between the two sets of data is quite remarkable, suggesting that indeed the experimentally observed trends are a manifestation of quantum confinement effects.

## 4. Conclusions

Ultrathin Pd films, originally prepared by frequency magnetron sputtering, were subsequently annealed at 530 °C in the presence of air, resulting in the creation of highly crystalline PdO thin films. Their absorbance spectra were experimentally studied, and their optical band gap was determined with the help of Tauc plots. The results revealed that as the film thickness was reduced all the way down to 0.7 nm, a significant “blue shift” of more than 0.5 eV was observed. Although this behavior by itself is highly indicative of quantum confinements effects, we further employed theoretical calculations capable of capturing such effects. The agreement with the experimental results proved to be quite accurate, offering an additional argument in favor of the quantum confinement interpretation.
